# XPO1/CRM1 is a promising prognostic indicator for neuroblastoma and represented a therapeutic target by selective inhibitor verdinexor

**DOI:** 10.1186/s13046-021-02044-z

**Published:** 2021-08-12

**Authors:** Lijia Pan, Cheng Cheng, Peiwen Duan, Kai Chen, Yeming Wu, Zhixiang Wu

**Affiliations:** 1 Department of Pediatric Surgery, Xinhua Hospital, School of Medicine, Shanghai Jiaotong University, Shanghai, 200092 China; 2 Division of Pediatric Oncology, Shanghai Institute of Pediatric Research, Shanghai, 200092 China; 3 Department of Pediatric Surgery, Children’s Hospital of Soochow University, Suzhou, 215003 China

**Keywords:** Neuroblastoma, XPO1, Verdinexor, KPT-335, P53, PI3K/AKT pathway

## Abstract

**Background:**

High-risk neuroblastoma patients have a 5-year survival rate of less than 50%. It’s an urgent need to identify new therapeutic targets and the appropriate drugs. Exportin-1 (XPO1), also known as chromosomal region maintenance 1, plays important roles in the progression of tumorigenesis. However, the prognostic and therapeutic values of XPO1 in neuroblastoma have not been reported.

**Methods:**

Correlations between XPO1 expression level and clinical characteristics were analyzed using the Neuroblastoma Research Consortium (NRC) dataset and tissue microarray analysis. Cell proliferation assays, colony formation assays, apoptosis assays, cell cycle analysis were performed to analyze the anti-tumor effects of verdinexor (KPT-335) in vitro. Western blot and mRNA sequencing were performed to explore underlying mechanism. In vivo anti-tumor effects of verdinexor were studied in a neuroblastoma xenograft model*.*

**Results:**

Higher XPO1 levels were associated with advanced stage and poor prognosis in neuroblastoma patients. The specific inhibitor of XPO1 verdinexor suppressed the neuroblastoma cell growth both in vitro and in vivo*.* Specifically, inhibition of XPO1 suppressed the neuroblastoma cell proliferation and induced cell apoptosis by nuclear accumulation of FOXO1 and RB1 in the neuroblastoma due to the inhibition of the PI3K/AKT pathway, and induced G0/G1 phase cell cycle arrest by activation of P53 function.

**Conclusions:**

XPO1 is a promising prognostic indicator for neuroblastoma and a novel target for antitumor treatment with selective inhibitor verdinexor.

**Supplementary Information:**

The online version contains supplementary material available at 10.1186/s13046-021-02044-z.

## Background

Neuroblastoma is the most common extracranial solid tumor in children and originates from the neural crest cells that give rise to the sympathetic nervous system [1, 2]. About 1.1 out of 100,000 children in the United States are diagnosed with neuroblastoma per year, accounting for 7% of all childhood malignancies [3]. Neuroblastomas show extensive heterogeneity in their natural history, ranging from spontaneous regression to rapid progression [4]. Low-risk and intermediate-risk patients demonstrate good survival, but the 5-year survival rate in high-risk patients remain less than 50% [5]. Although the introduction of dinutuximab and I-131 MIBG have improved response rates and cancer outcomes, some patients don’t respond well to current chemotherapeutic drugs [6], and the first-line chemotherapeutic drugs for childhood neuroblastoma remain unchanged for several years [5]. Therefore, it is important to identify new therapeutic targets and drugs for neuroblastoma.

Exportins belong to the karyopherins beta family and are responsible for the transportation of most proteins and RNA from the nucleus to the cytoplasm [7]. Exportin-1 (XPO1), also known as chromosomal region maintenance 1 (CRM1), is the most studied member of exportins involved in tumorigenesis [8]. XPO1 forms a hydrophobic groove through the HEAT repeats that can bind with proteins containing leucine-rich nuclear export signal [9]. XPO1 mediates the transport of 200 mammalian cargo proteins from the nucleus into the cytoplasm, including several oncoproteins (such as Snail, Survivin, PI3K, AKT)and tumor suppressor proteins (such as P53, P27, p21, RB, FOXOs) [10, 11]. Second-generation XPO1 inhibitors, also known as selective inhibitors of nuclear transport (SINE) compounds, have antitumor effects on hematologic malignancies, central nervous system tumors and solid tumors [12]. Mechanistically, SINE, including KPT-185, KPT-330 and KPT-335, inhibit XPO1 by promoting its degradation [13, 14]. Selinexor (KPT-330) is a safe SINE with a broad antitumor effect as reported in pre-clinical or clinical trials [15]. However, only a few studies have investigated the efficacy of XPO1 selective inhibitor verdinexor (KPT-335). Studies conducted in animals with stage I and II canine lymphoma confirmed that KPT-335 is safe and effective, and the adverse reactions (mild anorexia and vomiting) are treatable [16, 17]. The biological function of XPO1 in neuroblastoma and the therapeutic effect of the XPO1 inhibitor KPT335 in neuroblastoma have not yet been evaluated.

This study is the first to demonstrated the significant association between overexpression of XPO1 and poor clinical characteristics and prognosis in neuroblastoma patients. Verdinexor, the specific inhibitor of XPO1, suppressed the neuroblastoma cell growth and induced cell apoptosis by FOXO1 and RB1 nuclear accumulation through inhibition of the PI3K/AKT pathway. Verdinexor also induced the nuclear accumulation of P53 in neuroblastoma cells and induced G0/G1 phase cell cycle arrest by activating P53 function. In an in vivo study, verdinexor inhibited tumor growth without causing obvious toxic effects. Taken together, XPO1 was a promising prognostic indicator for neuroblastoma and a novel target for antitumor treatment with selective inhibitor verdinexor.

## Materials and methods

### Specimens and tissue microarray

Tissue samples were obtained from 64 neuroblastoma patients with pathologically confirmed neuroblastoma after radical resection at the Department of General Surgery, Xinhua Hospital, Shanghai Jiao Tong University School of Medicine (Shanghai, China) between 2012 and 2015. Ethical approval was obtained from the Ethics Committee of Xinhua Hospital, and informed consent was obtained from all patients. The age at diagnosis ranged from 2 to 156 months, and the male: female ratio was 7:9. Tissue microarray (TMA) was constructed using Servicebio (Wuhan, China) and scanned using Pannoramic MIDI [18] (3D HISTECH, Budapest, Hungary).

### Cell lines and reagents

The human neuroblastoma cell lines SK-N-BE(2) and SH-SY5Y were obtained from the Shanghai Cell Institute National Cell Bank. Cell line authentication by STR profiling was conducted by GENEWIZ, lnc. (Suzhou, China). All cells were maintained in Dulbecco’s Modified Eagle’s Medium (DMEM; HyClone, Logan, UT, USA) with 10% fetal bovine serum (FBS; Gibco, Grand Island, NY, USA) and placed in an incubator containing 5% CO_2_ humidified atmosphere at 37 °C. All experiments were performed with mycoplasma-free cells. KPT-335, idasanutlin (RG7388), and 740 Y-P were purchased from MedChemExpress (Monmouth Junction, NJ, USA). CHX and actinomycin D were purchased from BioVision (San Francisco, CA, USA). All reagents were dissolved in dimethyl sulfoxide using an appropriate stock concentration and diluted with medium before conducting the experiments.

### siRNA transfection

The siRNAs used to establish XPO1 knockdown cell lines were designed and synthesized using HuaGene Biotech (Shanghai, China). The siRNA-transfected neuroblastoma cells used were as follows: *XPO1*-siRNA1: 5- AUUCGACUUGCGUACUCAAAUTT-3, *XPO1*-siRNA2: 5- CCUGCUUUCAAGGAACAUUUATT-3′, and *XPO1*-siRNA3: 5′- GCUCAAGAAGUACUGACACAUTT-3′. *P53*-siRNA: 5- CGGCGCACAGAGGAAGAGAAUTT-3, *FOXO1*-siRNA: 5- CAUGGACAACAACAGUAAAUUTT-3, *RB1*-siRNA: 5- UCGCUUGUAUUACCGAGUAAUTT-3, SK-N-BE(2), and SH-SY5Y cells were transfected with target siRNA or negative control siRNA using the Lipofectamine™ RNAiMAX Transfection Reagent (Invitrogen, Carlsbad, CA, USA) according to the manufacturer’s protocol.

### Cell proliferation assays and colony formation assays

Cell proliferation assays were performed using the Cell Counting Kit-8 reagent (CCK-8; Dojindo, Japan). SK-N-BE(2) and SH-SY5Y cells were cultured in 96-well plates at a density of 2 × 10^4^ cells per well, and the absorbance value (OD) at 450 nm was measured using an automated microplate reader (Bio-Rad, Hercules, CA, USA) to assess the cell proliferation ability. In the colony formation assays, SK-N-BE(2) and SH-SY5Y cells were seeded in 6-well plates at a density of 1 × 10^4^ cells per well for one week. At the end of the procedure, the cells were fixed with 4% paraformaldehyde for 15 min and then stained with 0.5% crystal violet (Beyotime, Shanghai, China). The number of colonies was counted and examined under a microscope (Leica, Solms, Germany).

### Apoptosis assays

An Annexin V-FITC Apoptosis Detection Kit (Beyotime, Shanghai, China) was used to conduct the apoptosis assay. Cells treated with KPT-335 for 48 h were collected from the culture medium and washed three times with PBS. Next, 5 μL of FITC, 5 μL of propidium iodide (PI) (50 μg/mL), and 200 μL of Annexin V binding buffer (1 ×) were added to the mixture. Then, the cell suspension was incubated in the dark at room temperature for 15 min. Apoptosis analysis was performed using FACSCalibur (BD Biosciences, San Diego, CA, USA).

### Cell cycle assays

Cell Cycle Analysis Kit (Beyotime, Shanghai, China) was used to conduct cell cycle assays. Cells treated with KPT-335 for 48 h were collected from the culture medium, washed with PBS three times, and fixed with 70% ethanol at 4 °C overnight. Then, the cells were collected (1,000 g, 5 min), resuspended in PBS with 1 ml/mL propidium and 10 mg/mL RNase, and incubated for 30 min at 37 °C. Cell cycle analysis was performed using FACSCalibur.

### TdT-mediated dUTP nick-end labeling assays

A One Step TUNEL Apoptosis Assay Kit (Beyotime, Shanghai, China) was used to conduct TdT-mediated dUTP nick-end labeling (TUNEL) assays. After treatment with KPT-335 for 48 h, the cells were fixed with 4% paraformaldehyde for 15 min and then treated with PBS with 0.3% Triton X-100 for 5 min. The cells were treated with TUNEL test solution according to the manufacturer’s protocol and examined under a fluorescence microscope (Leica, Solms, Germany).

### Quantitative real-time PCR

Total RNA was extracted from SK-N-BE(2) and SH-SY5Y cells using TRIzol (Takara, Japan) according to the manufacturer’s instructions. cDNA was synthesized from 1 μg of total RNA using PrimeScript Reverse Transcriptase (Takara, Japan). The primers used for amplification were as follows: *GAPDH* forward primer (5-CAACAGCCTCAAGATCATCAGC-3), *GAPDH* reverse primer (5-TTCTAGACGGCAGGTCAGGTC-3), *P53* forward primer (5- CAGCACATGACGGAGGTTGT-3), *and P53* reverse primer (5- TCATCCAAATACTCCACACGC-3). Quantitative real-time PCR was conducted using the StepOnePlus™ Real-Time PCR system (Applied Biosystems), and *P53* expression levels were detected using the SYBR-Green method (Takara, Japan).

### Western blot

RIPA buffer (Cell Signaling, Danvers, MA, USA) was used to extract total protein from cells. Cell fraction lysates were extracted using nuclear and cytoplasmic extraction reagents (Thermo Fisher Scientific, Waltham, MA, USA) according to the manufacturer’s protocol. Next, proteins were separated by sodiumdodecyl sulfate–polyacrylamide gel electrophoresis (SDS-PAGE) and transferred onto PVDF membranes (Millipore, MA, USA). Then, 5% skim milk was used to block the blots for 2 h at room temperature. A series of primary antibodies were added to the appropriate position of the PVDF membranes and incubated overnight at 4 °C. Antibodies against XPO1, Bad, Bax, Bcl-2, P53, P27, P21, PI3K, p-PI3K (Tyr458), AKT, p-AKT (Ser473), FOXO1, p-FOXO1 (Ser256), RB1, p-RB1 (Ser608), H3, GAPDH, and β-actin (rabbit, 1:1,000) were purchased from Cell Signaling Technology (Danvers, MA, USA). Finally, all blots reacted with the suitable HRP-conjugated secondary antibody (Beyotime, Shanghai, China) and analyzed using BIO-RAD ChemiDoc XRS + (Bio-Rad, Hercules, CA, USA).

### mRNA Sequencing and bioinformatics analysis

mRNA sequencing and bioinformatics analysis of SK-N-BE(2) treated with KPT-335 for 24 h were conducted by the Beijing Genomics Institute (Beijing, China). Raw data were submitted to GEO (GSE163987). Briefly, after filtering the sequencing data using SOAPnuke (v1.5.2) [18], clean reads were obtained in FAtSTQ format. Then, HISAT2 (v2.0.4) [19] was used to map clean reads to the reference genome, while Bowtie2 (v2.2.5) [20] was used to align clean reads to the reference coding gene set. Gene expression levels were analyzed using RSEM (v1.2.12) [21], while differential gene expression was analyzed using DESeq2(v1.4.5) [22] under the condition of |Log_2_FC|≥ 1,6 and *p* < 0.001. Based on differential gene expression, pheatmap (v1.0.8) [23] was used to draw a heatmap. GO analysis (http://www.geneontology.org/) and KEGG analysis (https://www.kegg.jp/) were conducted using the hypergeometric test with Phyper (https://en.wikipedia.org/wiki/Hypergeometric_distribution).

### Immunofluorescence and Immunohistochemistry

Immunofluorescence (IF) staining and immunohistochemistry (IHC) staining were conducted using the Servicebio Technology (Wuhan, China). The IHC staining results were evaluated using a semiquantitative histologic scoring system (H-score). The positive neoplastic cell percentage and staining intensity degree were both considered to calculate the H-score. The intensity was scored on a scale of 0 to 3 (0, negative; 1, weak; 2, medium; and 3, strong). The H-score was calculated using the following formula: H-score = ∑(Pi × I) = (percentage of cells with weak intensity × 1) + (percentage of cells with moderate intensity × 2) + percentage of cells with strong intensity × 3) [24], where Pi is the percentage of positive tumor cells and I is the staining intensity.

### Xenograft model study

Male nude mice were purchased from the Shanghai Laboratory Animal Center of the Chinese Academy of Sciences (Shanghai, China). The in vivo study was approved by the Ethics Committee of Xinhua Hospital. To establish a subcutaneous xenograft model, SK-N-BE(2) cells (5 × 10^6^ cells in 100 µL PBS) were injected subcutaneously in two groups of nude mice (5 mice/group). The two groups were treated via oral gavage three times per week: vehicle (0.3% CMC-Na) and KPT-335 (10 mg/mg) dissolved in vehicle. Tumor volumes (1/2 × width^2^ × length) were measured weekly using a caliper. After four weeks of treatment, the xenograft tumors were excised and weighed.

### Statistical analysis

All statistical analyses were conducted using Prism 8 software (GraphPad Software, Inc., La Jolla, CA, USA) and SAS v8.0 (SAS Institute Inc., Cary, NC, USA). The best cut-off value for *XPO1* mRNA expression level and protein level were determined using the X-tile software (best *p* value) [25]. The association between XPO1 and clinical characteristics in neuroblastoma was analyzed using Pearson’ s chi-square test. Prognostic analysis was performed using univariate and multivariate Cox regression models. The log-rank (Mantel-Cox) test was used for overall survival analysis. The Student’s t-test was used for statistical analysis, and nonparametric tests were conducted when necessary. All assays were conducted independently at least three times. Results were expressed as mean ± standard deviation (SD) and considered significant when the *p* value is < 0.05.

## Results

### Overexpression of XPO1 is associated with poor clinical characteristics and prognosis of neuroblastoma patients

**Fig. 1:**
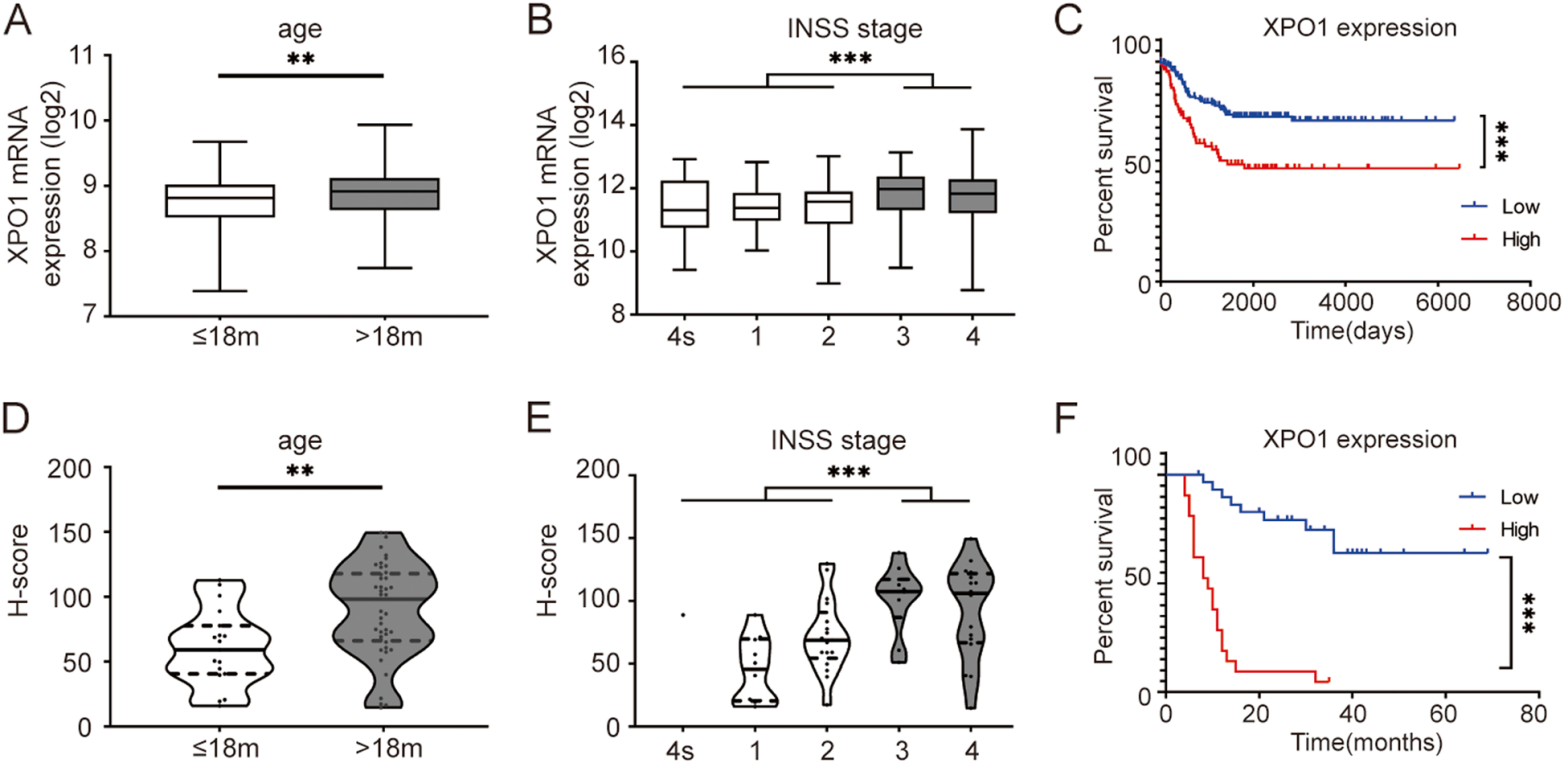
Overexpression of XPO1 is associated with poor clinical characteristics and prognosis of neuroblastoma patients. **A***XPO1* mRNA is overexpressed in patients with age at diagnosis more than 18 months and **B** advanced INSS stage. C Patients with high *XPO1* mRNA expression present shorter overall survival time. **D** panoramic view of TMA. **E** XPO1 protein is overexpressed in patients with age at diagnosis more than 18 months and **F** advanced INSS stage. **G** Patients with high XPO1 protein expression present shorter overall survival time. TMA, specimens and tissue microarray. INSS, International Neuroblastoma Staging System. ** *P* < 0.01, *** *P* < 0.001

First, the correlation between *XPO1* mRNA expression level and clinical characteristics was analyzed using the NRC dataset (GSE85047). Patients with high-risk clinical parameters presented higher *XPO1* expression, including age at diagnosis > 18 months and advanced INSS stage (Fig. 1A, B, *p* < 0.05). Then, a subgroup analysis was performed to further clarify the relationship between the mRNA expression level of *XPO1* and overall survival. Patients who showed *XPO1* overexpression had shorter overall survival (Fig. 1C, *p* < 0.05).

Next, the correlation between XPO1 protein expression level and clinical characteristics was analyzed according to the results of the TMA analysis. TMA was scanned using Pannoramic MIDI (Fig. 1D), and the clinical information of 64 samples were retrieved. Consistent with the above results, patients diagnosed at the age of 18 months or older and with advanced INSS stage had higher XPO1 expression (Fig. 1E, F, *p* < 0.05). According to the results of the subgroup analysis, patients with higher XPO1 expression had shorter overall survival (Fig. 1G, *p* < 0.05). XPO1 overexpression was associated with several high-risk parameters, such as age at diagnosis (18 months or older) and advanced INSS stage (Table 1, *p* < 0.05). Univariate and multivariate analyses suggested that XPO1 expression was a poor independent prognosis factor (Table 2, *p* = 0.003).

**Table 1:** Tissue microarray identifies the association between XPO1 expression and clinical characteristics in neuroblastoma patients

Factors	Case No	XPO1 expression		*P* value
		low, No. (%)	high, No. (%)	
Gender				0.181
Male	28	16 (57.1)	12 (42.9)	
Female	36	27 (75.0)	9 (25.0)	
Age (month)				0.019
≤ 18	19	17 (89.5)	2 (10.5)	
> 18	45	26 (57.8)	19 (42.2)	
MYCN amplification				0.016
No	35	28 (80.0)	7 (20.0)	
Yes	20	9 (45.0)	11 (55.0)	
Tumor stage (INSS)				< 0.001
4 s + 1 + 2	29	27 (93.1)	2 (6.9)	
3 + 4	26	10 (38.5)	16 (61.5)	
Pathological histotype				0.580
Neuroblastoma	23	14 (60.9)	9 (39.1)	
Ganglioneuroblastoma	41	29 (70.7)	12 (29.3)	
Preoperative chemotherapy				0.008
No	31	26 (83.9)	5 (16.1)	
Yes	33	17 (51.5)	16 (48.5)	
Death				< 0.001
No	23	22 (95.7)	1 (4.3)	
Yes	29	9 (31.0)	20 (69.0)	

Pearson’s chi-squared test and CMH test was used for statistical analysis; *INSS* International Neuroblastoma Staging System

**Table 2:** Cox proportional hazards model for prognostic factors analysis in neuroblastoma patients (TMA)

Variables	Favorable/Unfavorable	Univariate analysis		Multivariate analysis	
		HR (95% CI)	*P* value	HR (95% CI)	*P* value
Gender	female /male	0.893 (0.430–1.856)	0.762		
Age (month)	≤ 18/ > 18	2.549 (1.083–5.997)	0.032	2.029 (0.778–5.289)	0.148
Pathological histotype	ganglioneuroblastoma/ neuroblastoma	0.538 (0.249–1.161)	0.114		
MYCN amplification	no/yes	6.973 (2.880–16.878)	< 0.001	3.299 (1.097–9.923)	0.034
Preoperative chemotherapy	no/yes	5.219 (2.200–12.382)	< 0.001	1.144 (0.297–4.406)	0.845
Tumor stage (INSS)	4 s + 1 + 2/3 + 4	8.691 (3.397–22.237)	< 0.001	2.676 (0.780–9.180)	0.118
XPO1 expression	low/high	10.652 (4.330–26.204)	< 0.001	4.392 (1.641–11.754)	0.003

Cox regression was used for statistical analysis. *95%CI* 95% confidence interval, *HR *Hazard ratio, *INSS *International Neuroblastoma Staging System

### XPO1 inhibitor verdinexor blocks the proliferation of neuroblastoma cells by promoting cell apoptosis

**Fig. 2:**
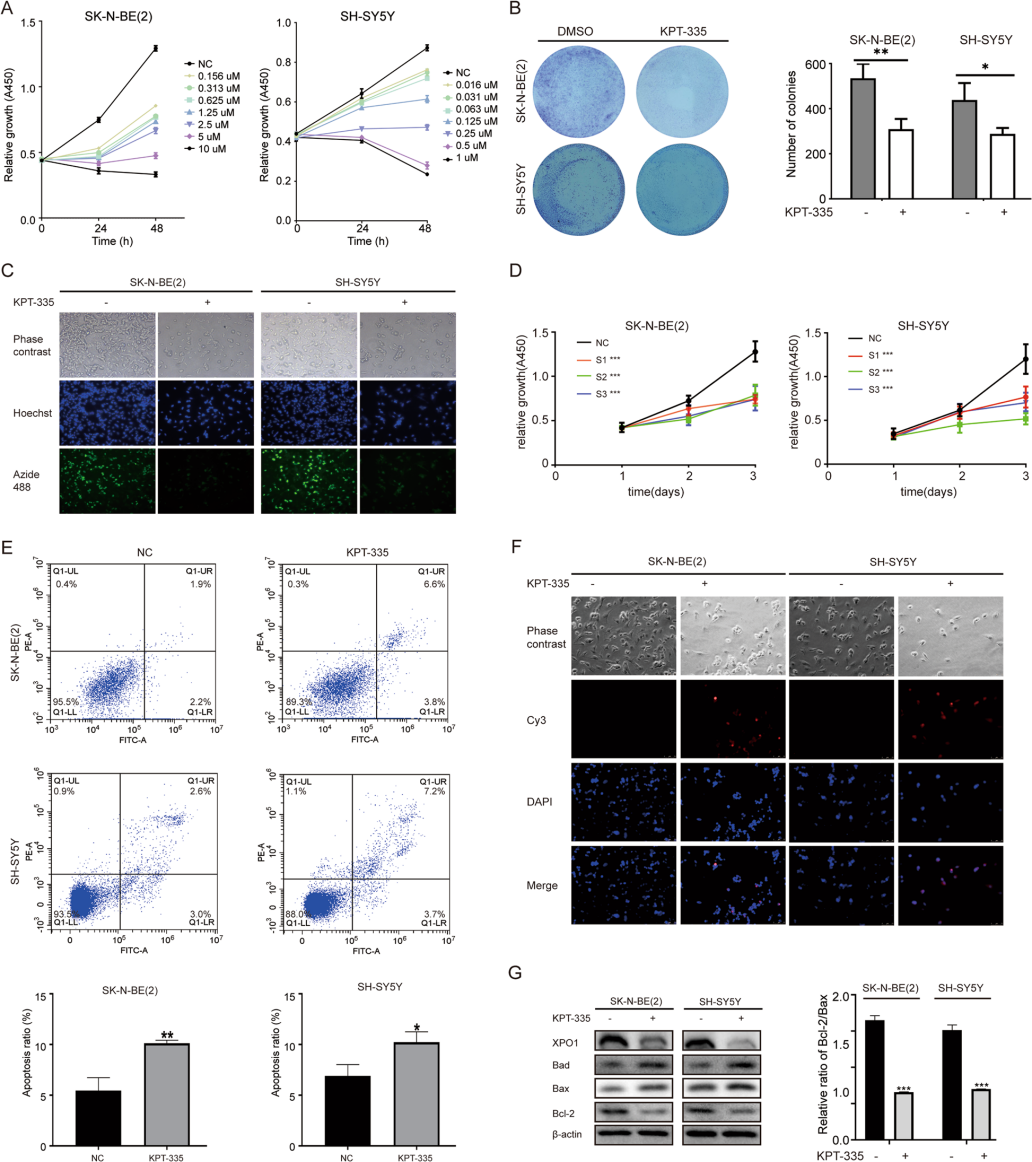
XPO1 inhibitor KPT-335 inhibits neuroblastoma cells proliferation by promoting cell apoptosis. **A** Proliferation inhibition of SK-N-BE(2) and SH-SY5Y by selective inhibitor KPT-335 in dose and time-dependent manners. **B** Inhibition of colonies formation after KPT-335 treatment in SK-N-BE(2) and SH-SY5Y. **C** Fluorescence images of EdU-488 after KPT-335 treatment in SK-N-BE(2) and SH-SY5Y. **D** knockdown of XPO1 by siRNA inhibited SK-N-BE(2) and SH-SY5Y proliferation. **E** KPT-335 induced SK-N-BE(2) and SH-SY5Y cells apoptosis. **F** Fluorescence images of TUNEL after KPT-335 treatment in SK-N-BE(2) and SH-SY5Y. **G** Bad, Bax, Bcl-2 and XPO1 expression levels detected by western blotting. TUNEL, TdT-mediated dUTP Nick-End Labeling assays. Data presented as mean ± SD (*n* = 3). * *P* < 0.05, ** *P* < 0.01, *** *P* < 0.001

To evaluate the function of XPO1 in neuroblastoma cells, cell viability was determined by conducting the CCK-8 assay. Results showed that the XPO1 selective inhibitor verdinexor (KPT-335) significantly inhibited neuroblastoma cell proliferation in a dose-and time-dependent manner (Fig. 2A). Verdinexor IC50 values for SK-N-BE(2) and SH-SY5Y cells were approximately 1.4 µM and 0.3 µM at 48 h, respectively, and were selected for subsequent experiments. Colony formation assays were conducted to study the colony forming capacity of single cells. In colony formation assays, the change of size and number of colonies was visible in the photos, and the number of colonies could be quantified. KPT-335 significantly reduced the number and size of colonies formed by neuroblastoma cells (Fig. 2B). As shown in Fig. 2C, the EdU-488 assay confirmed that KPT-335 significantly suppressed the proliferation of SK-N-BE(2) and SH-SY5Y cells. To confirm whether this antitumor effect was mediated by XPO1 inhibition rather than an off-target effect, a repeat growth assay was performed by treating the cells with three *XPO1*-siRNAs. XPO1 was efficiently knocked down by XPO1-siRNAs in protein level (Figure S1A). Results confirmed that the inhibition of XPO1 suppressed the proliferation of SK-N-BE(2) and SH-SY5Y cells (Fig. 2D). In addition, results of flow cytometry assay showed that the apoptotic rate was significantly increased in KPT-335-treated SK-N-BE(2) and SH-SY5Y cells compared with the negative control cells (Fig. 2E). TUNEL assays confirmed that KPT-335 induced apoptosis in SK-N-BE(2) and SH-SY5Y cells (Fig. 2F). Moreover, apoptosis-related Bcl-2 family proteins were detected by conducting the Western blot analysis. Results suggested that the expression of Bad and Bax was significantly increased, while the expression of Bcl-2 was significantly reduced, especially the ratio between Bcl-2 and Bax (Fig. 2G). These results indicated that KPT-335 significantly suppressed the proliferation of neuroblastoma cells and induced cell apoptosis in vitro.

### XPO1 inhibitor verdinexor inducing nuclear accumulation of FOXO1 and RB1 by inhibiting the PI3K/AKT pathway

**Fig. 3:**
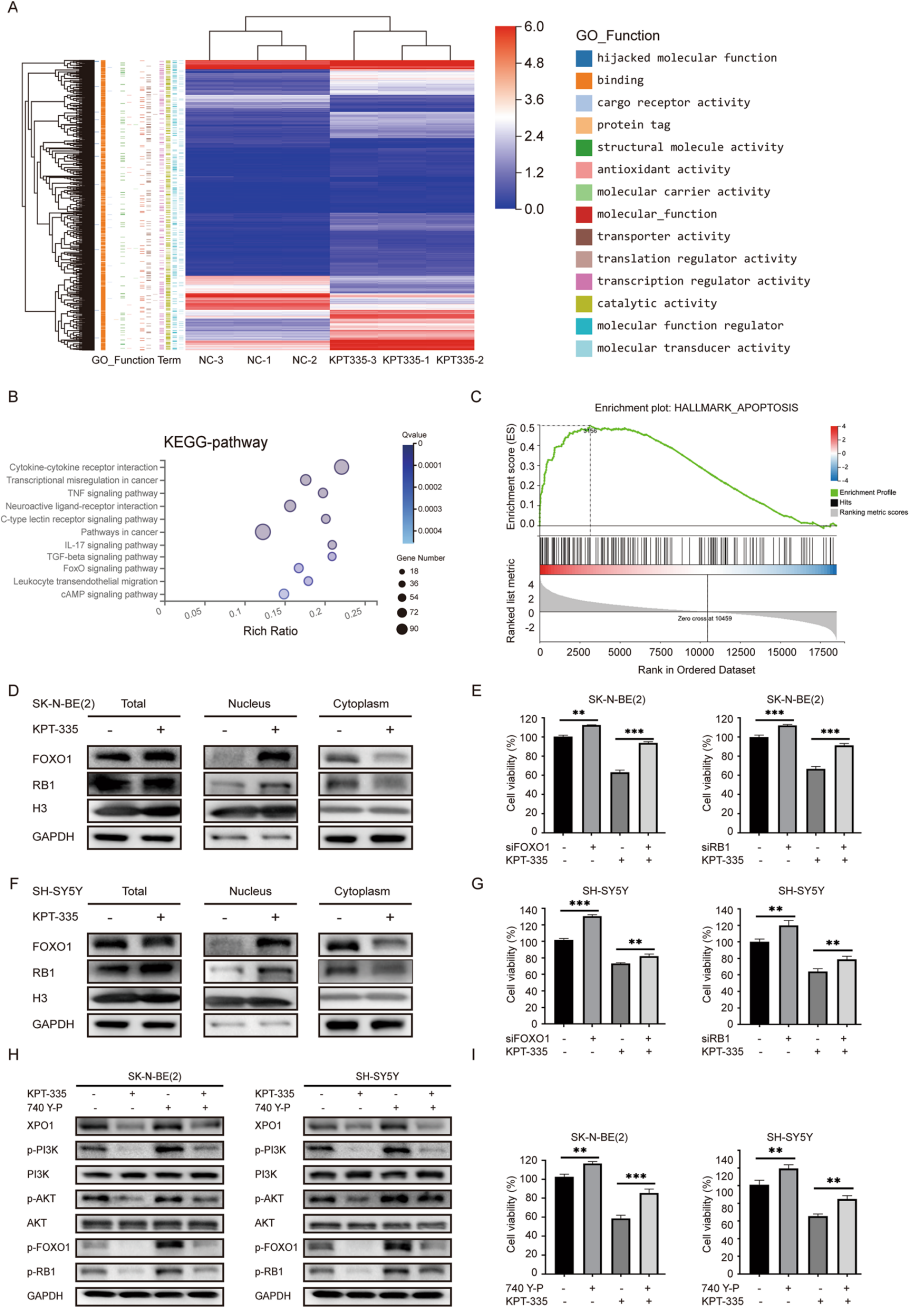
XPO1 inhibitor KPT-335 induces nuclear accumulation of FOXO1 and RB1 through inhibiting PI3K/AKT pathway. **A** Heatmap and Go function analysis, **B** KEGG pathway, **C** GSEA analysis of differential genes (|Log2FC|≥ 1.6, *P* < 0.001) of SK-N-BE(2) after KPT-335 treatment. **D** Western blot analysis of FOXO1 and Rb1 levels in nucleus and cytoplasm of SK-N-BE(2) after KPT-335 treatment. **E** knockdown of FOXO1 and RB1 attenuated effective of KPT-335 in SK-N-BE(2). **F** Western blot analysis of FOXO1 and RB1 levels in nucleus and cytoplasm of SH-SY5Y after KPT-335 treatment. **G** Knockdown of FOXO1 and RB1 attenuated inhibition effects of KPT-335 in SH-SY5Y. **H** XPO1, p-PI3K, PI3K, p-AKT, AKT, p-FOXO1, p-RB1 expression levels detected by western blotting. **I** 740 Y-P attenuated inhibition effects of KPT-335 in SK-N-BE(2) and SH-SY5Y. Data presented as mean ± SD (*n* = 3). ** *P* < 0.01, *** *P* < 0.001

To further examine the mechanism of KPT-335 in neuroblastoma cells, mRNA-sequencing of SK-N-BE(2) cells was performed after treatment with KPT-335. Under the condition of |Log_2_FC|≥ 1,6 and *p* < 0.001, 1,495 genes were upregulated and 476 genes were downregulated. The enrichment analysis and GO function analysis of these differential genes showed that most of the differential genes were related to the “binding” function of XPO1 (Fig. 3A). KEGG analysis indicated significant changes in the “FoxO signaling pathway” (Fig. 3B). Consistent with the previous results, the GSEA analysis indicated significant changes in the “apoptosis” pathway (Fig. 3C). Moreover, FOXO1 and RB1 were considered as cargo proteins of XPO1 [10, 11]. Western blot analysis of cell fractionated lysates confirmed that the overall expression levels of FOXO1 and RB1 proteins did not change significantly after KPT-335 treatment, but they were significantly accumulated in the nucleus and decreased in the cytoplasm (Fig. 3D and F). In addition, FOXO1 and RB1 was efficiently knocked down by siRNA in protein level (Figure S1 B). Knockdown of FOXO1 and RB1 limited the antitumor effects of KPT-335 in SK-N-BE (2) and SH-SY5Y cells (Fig. 3E and G). It has been reported that AKT directly phosphorylates FOXO1 and promotes its exportation from the nucleus [26]. As shown in Fig. 3H, KPT-335 significantly suppressed the phosphorylation levels of PI3K, AKT, FOXO1, and RB1 in SK-N-BE (2) and SH-SY5Y cells, while PI3K agonist 740Y-P partially rescued this effect. In addition, 740Y-P limited the antitumor effect of KPT-335 in neuroblastoma cells (Fig. 3I). These results suggest that KPT-335 exerts antitumor effects in neuroblastoma cells by inducing nuclear accumulation of FOXO1 and RB1 through inhibition of the PI3K/AKT pathway.

### XPO1 inhibitor verdinexor inducing the nuclear accumulation of P53 in neuroblastoma cells

**Fig. 4:**
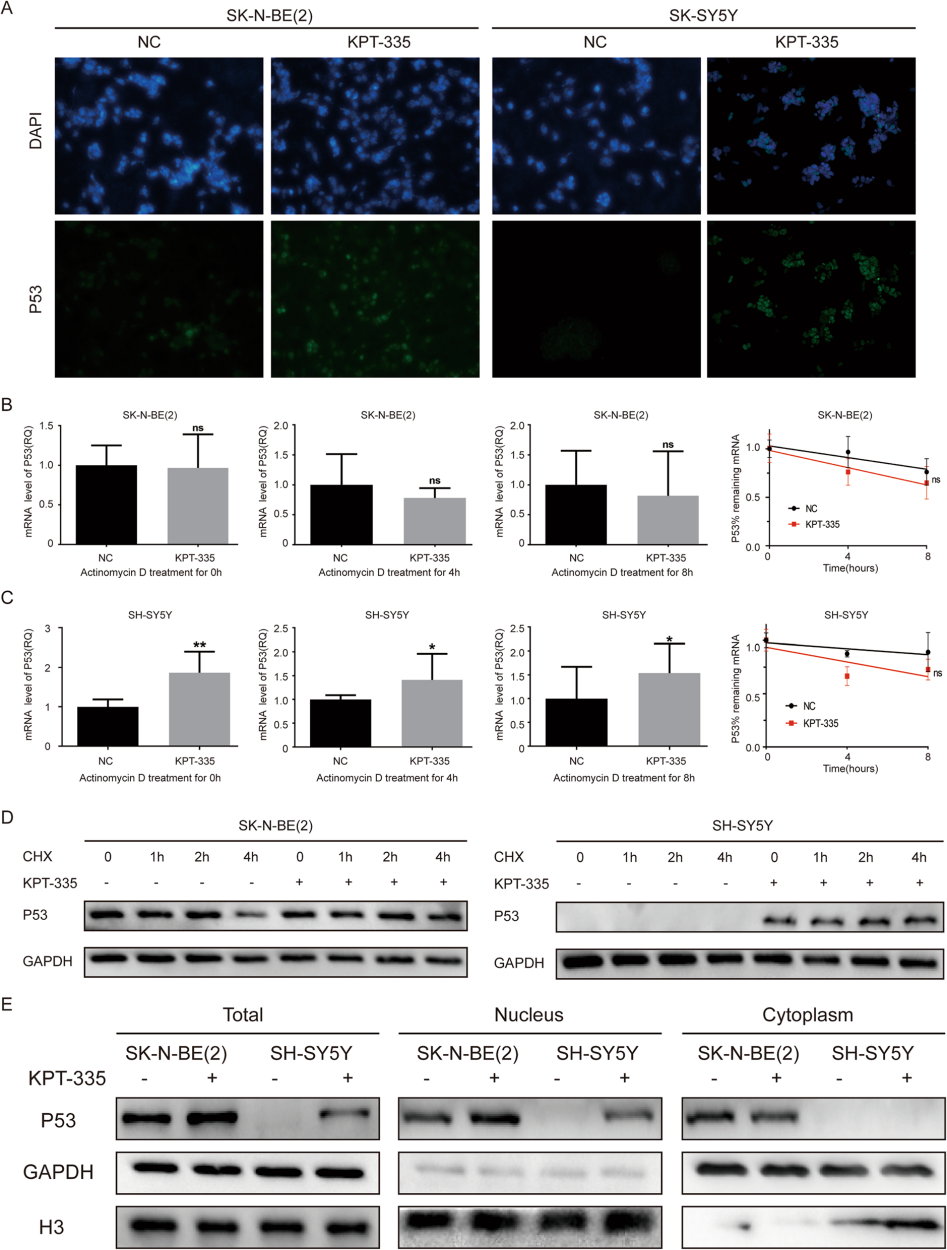
XPO1 inhibitor KPT-335 induces nuclear accumulation of P53 in in neuroblastoma cells. **A** Immunofluorescence images of nuclear P53 in SK-N-BE(2) and SH-SY5Y after KPT-335 treatment. **B** and **C** Degradation rate of *P53* mRNA in SK-N-BE (2) and SH-SY5Y after KPT-335 treatment. **D** Degradation rate of P53 protein in SK-N-BE (2) and SH-SY5Y after KPT-335 treatment. **E** Western blot analysis of P53 levels in nucleus and cytoplasm of SK-N-BE (2) and SH-SY5Y after KPT-335 treatment. Data presented as mean ± SD (*n* = 3). * *P* < 0.05, ** *P* < 0.01

P53 is the cargo protein of XPO1 and plays important roles in cell apoptosis [27]. As shown in Fig. 4A, immunofluorescence staining confirmed that KPT-335 induced the nuclear accumulation of P53 in both SK-N-BE(2) and SH-SY5Y cells. To further understand how KPT-335 increased P53 protein expression levels in SK-N-BE(2) and SH-SY5Y cells, we blocked the synthesis of *P53* mRNA with actinomycin D in SK-N-BE(2) and SH-SY5Y cells for 0 h, 4 h, and 8 h after KPT-335 treatment. KPT-335 only increased the *P53* mRNA expression in SH-SY5Y cells, but did not increase the *P53* mRNA expression in SK-N-BE(2) cells. KPT-335 did not affect the degradation rate of *P53* mRNA in SK-N-BE(2) or SH-SY5Y cells (Fig. 4B and C). Then, we blocked the translation or synthesis of P53 protein with CHX in SK-N-BE(2) and SH-SY5Y cells within 0 h, 1 h, 2 h, and 4 h after KPT-335 treatment. KPT-335 significantly reduced the degradation rate of P53 protein in SK-N-BE(2) and SH-SY5Y cells (Fig. 4D). Western blot analysis of cell fractionated lysates (cytoplasmic and nuclear extracts) also confirmed that KPT-335 increased the P53 protein expression in SK-N-BE(2) and SH-SY5Y cells, only in the nucleus as no changes in p53 levels in the cytoplasm are observed (Fig. 4E). These results demonstrated that KPT-335 reduced the degradation of P53 protein and induced P53 nuclear accumulation in neuroblastoma cells.

### XPO1 inhibitor verdinexor inducing G0/G1 phase cell cycle arrest by activating P53 function

**Fig. 5:**
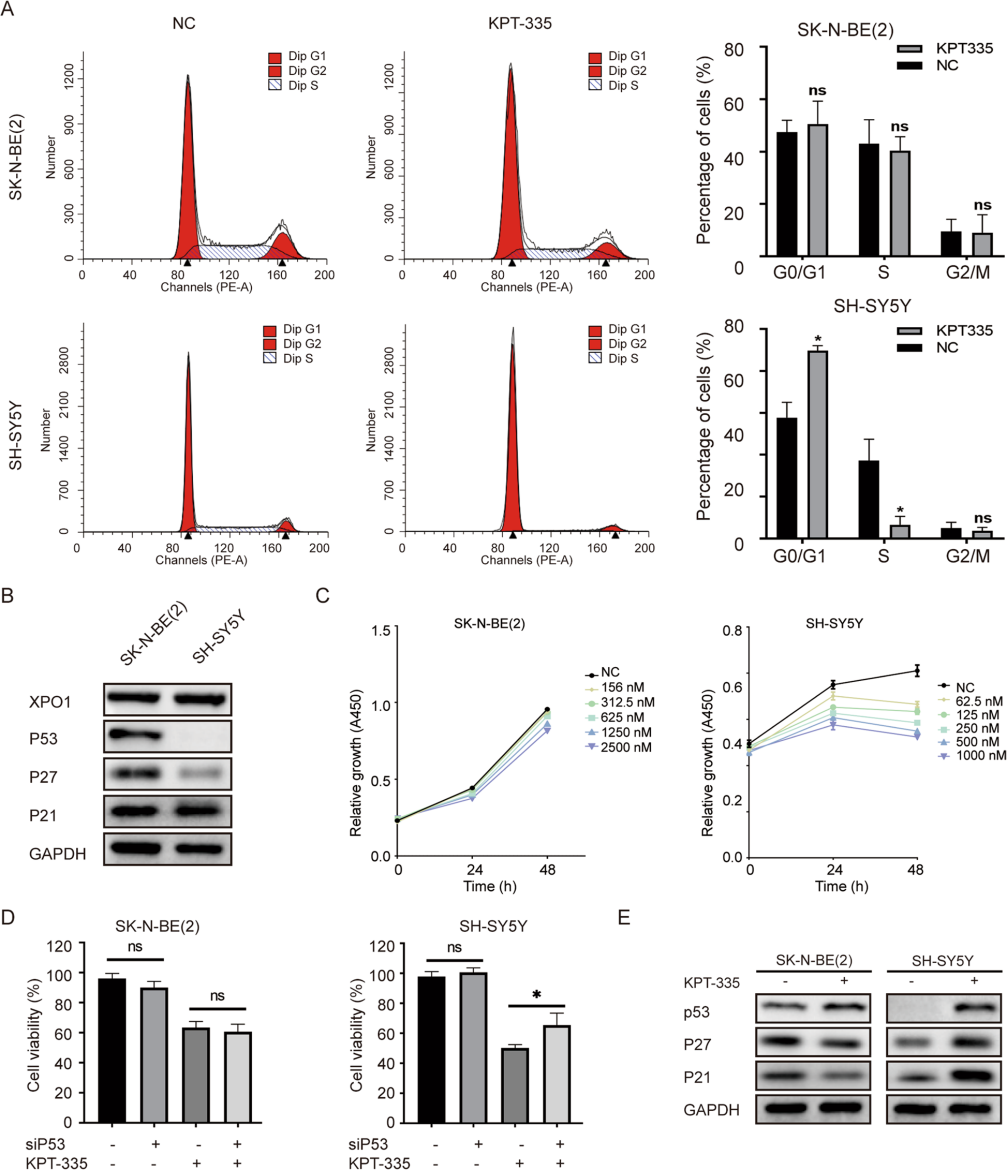
XPO1 inhibitor KPT-335 induces G0/G1 phase cell cycle arrest by activating P53 function. **A** KPT-335 induced G0/G1 phase cell cycle arrest in SH-SY5Y. **B** baseline of P53, P27, P21 in SK-N-BE(2) and SH-SY5Y. **C** Relative growth of SK-N-BE(2) and SH-SY5Y after RG7388 treatment. **D** Knockdown of *P53* attenuated inhibition effects of KPT-335 in SH-SY5Y. **E** P53, P27, P21 expression levels detected by western blotting after KPT-335 treatment. Data presented as mean ± SD (*n* = 3). * *P* < 0.05

Our study demonstrated that KPT-335 induces nuclear accumulation of P53 in neuroblastoma cells. However, KPT-335 only blocked the cell cycle progression in SH-SY5Y cells by increasing the G0/G1 phase fraction. No significant effect on the cell cycle changes of SK-N-BE (2) was observed (Fig. 5A). SK-N-BE (2) was recognized as an P53 mutant neuroblastoma cell. Although mutant P53 was highly expressed under physiological conditions in SK-N-BE (2), it was not functional [28]. As shown in Fig. 5B, Western blot analysis showed the expression of P53 and its downstream cycle-related proteins P27 and P21 in SK-N-BE(2) and SH-SY5Y cells. However, only SH-SY5Y cells were sensitive to the P53 agonist RG7388, while SK-N-BE (2) was almost ineffective (Fig. 5C), which was consistent with the results of a previous study [29]. P53 was efficiently knocked down by siRNA in protein level (Figure S1 C). Knockdown of P53 expression partially rescued the antitumor effect of KPT-335 in SH-SY5Y, but this effect was not observed in SK-N-BE(2) cells (Fig. 5D). Western blot analysis confirmed that KPT-335 increased the expression of P53 protein in SK-N-BE(2) and SH-SY5Y cells, but its downstream cycle-related proteins P27 and P21 were only increased in SH-SY5Y cells (Fig. 5E). These results indicated that KPT-335 induced G0/G1 phase cell cycle arrest in neuroblastoma cells by activating the P53 function.

### XPO1 inhibitor verdinexor inhibiting tumor growth in vivo

**Fig. 6:**
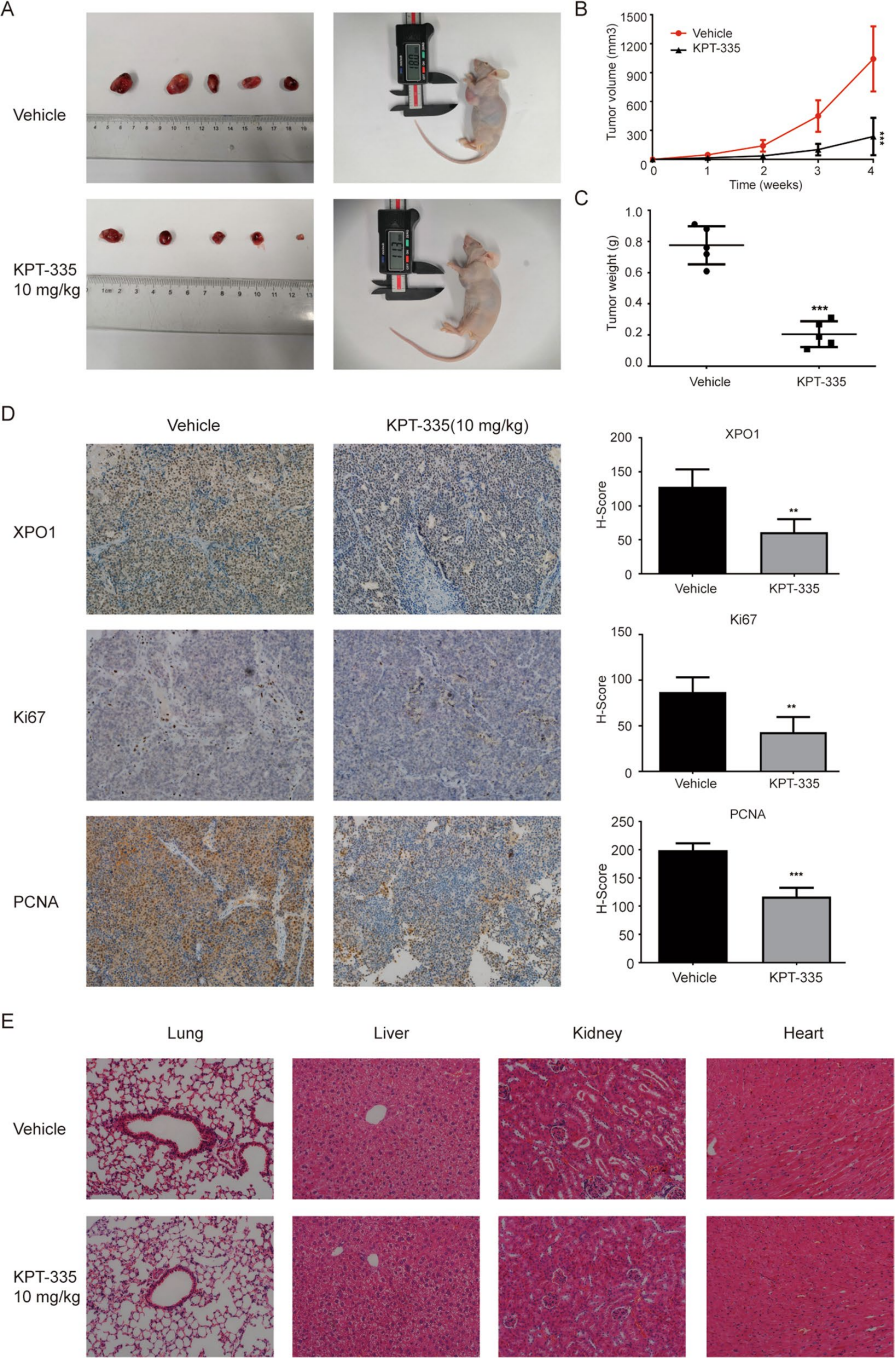
XPO1 inhibitor KPT-335 inhibits tumor growth in vivo. **A** photograph of sacrificed nude mice and xenograft. **B** Tumor volume and **C** weight of nude mice was reduced after KPT-335 treatment. **D** Immunohistochemistry showed XPO1, Ki67 and PCNA expression levels of xenograft tumor tissues was reduced (× 200). **E** No obvious pathological changes were found in the lung, liver, kidney and heart after KPT-335 treatment. Data presented as mean ± SD (*n* = 5). ** *P* < 0.01, *** *P* < 0.001

We constructed a nude mouse tumor model by injecting SK-N-BE(2) cells subcutaneously and then treated the nude mice with KPT-335 via oral gavage. Tumor volume and weight were significantly reduced compared with the control group (Fig. 6A–C). Immunohistochemical staining of tumor tissues showed that the expression of XPO1 protein in the treatment group was significantly reduced. Ki67 and PCNA, which are typical indicators of neuroblastoma proliferation, were also significantly reduced (Fig. 6D). In addition, there were no obvious pathological changes in the main organs (lung, liver, kidney, and heart) of nude mice after KPT-335 treatment (Fig. 6E). These results indicated that KPT-335 inhibited tumor growth in vivo without obvious toxic effects.

## Discussion

XPO1 inhibitor Leptomycin B (LMB) decreased the phosphorylation level of FOXO3a via a PI3K-dependent pathway to suppress neuroblastoma cell growth [30]. A previous study reported that the second generation XPO1 inhibitor KPT-330 has antitumor effects on neuroblastoma animal models [31]. These results suggest that XPO1 plays an important role in the tumorigenesis of neuroblastoma. This study was the first to demonstrate the significant association of XPO1 overexpression with poor clinical characteristics and prognosis in neuroblastoma patients. In an in vitro study, a specific XPO1 inhibitor, verdinexor, induced cell cycle arrest and apoptosis in neuroblastoma cells SH-SY5Y. As P53 wild type cells, SH-SY5Y induced cell cycle arrest by activating the P53 function. For P53 mutation neuroblastoma cells SK-N-BE(2), the anti-tumor effects of verdinexor were results of apoptosis rather than cell cycle arrest.

XPO1 is the key exporter of multiple tumor-suppressor proteins, including FOXOs, RB1, P53, P21, P27, and survivin [32]. Inhibition of XPO1 would perturb many of the hallmark pathways of tumorigenesis, thus exerting antitumor effects in broad malignancies. XPO1 inhibitor induced apoptosis in triple-negative breast cancer by promoting survivin nuclear accumulation and decreasing survivin cytoplasmic protein levels through repression of survivin transcription [33]. In ovarian carcinoma, XPO1 inhibitor inhibited tumor growth and improved the efficacy of cisplatin by nuclear accumulation of FOXO1 [34]. The process of transportation of target proteins by XPO1 might be regulated by the AKT pathway. Inhibition of XPO1 decreased phosphorylation of AKT (ser473) in tamoxifen-resistant breast cancer cells MCF-7 and tumor xenografts [35]. In addition, the transportation of FOXO1 and RB1 proteins out of the nucleus by XPO1 is dependent on the phosphorylation of AKT [36]. In another words, the hypophosphorylated FOXO1 and RB1 were difficult to transport out of the nucleus by XPO1. This indicated that the AKT signaling pathway was involved in the transportation of FOXO1 and RB1 by XPO1.Our research confirmed that verdinexor inhibited neuroblastoma proliferation and induced apoptosis through nuclear accumulation of FOXO1 and RB1. Verdinexor decreased the phosphorylation of PI3K and AKT, while PI3K agonist 740Y-P partially rescued this effect. Specifically, the inhibition of XPO1 reduced the phosphorylation levels of FOXO1 and RB1 by inhibiting the PI3K/AKT pathway.

P53 was the most studied cargo protein of XPO1 [37–40]. In these studies, the inhibition of XPO1 induced the restoration of nuclear P53 and, more importantly, activated the functions of downstream cycle-related proteins of the P53 pathway, including p21 and P27. Therefore, this effect mainly existed in P53 wild-type tumors. Would P53 mutation affect the anti-tumor effects of XPO1 inhibitors? Expression of p53 mutation significantly reduced the cytotoxicity of selinexor in refractory/relapsed diffuse large B-cell lymphoma cell lines [41]. In this case, the anti-tumor effects of XPO1 mainly depended on the function of P53, and P53 mutation would lead to strong drug resistance. However, for human fibrosarcoma cells HT-1080 and breast cancer cells MCF7, loss of p53 function altered cell cycle arrest after inhibition of XPO1 but didn’t weaken the anti-tumor effects [42]. This might be XPO1 strengthened the regulation of other anti-cancer targets or pathways in the absence of P53 function. Neuroblastoma was a predominantly P53 wild-type tumor. Only < 2% of neuroblastoma patients presented with p53 mutations at diagnosis [43]. In our study, KPT-335 induced G0/G1 phase cell cycle arrest in neuroblastoma cells by restoring nuclear P53. This effect was not observed in SK-N-BE(2) cells due to the mutation of the P53 gene. However, regardless of the presence of the P53 mutation, XPO1 inhibition exerted significant antitumor effects in neuroblastoma cells. This could be explained by the inhibition of XPO1 interference with many of the hallmark pathways in neuroblastoma. FOXO1, RB1, and P53 were the key proteins in our study.

LMB, a first-generation XPO1 inhibitor, failed to show efficacy in patients in a clinical trial due to the occurrence of permanent XPO1 inhibition-mediated intolerable cytotoxicity [44]. The second-generation XPO1 inhibitors, such as KPT-185, KPT249, KPT-251, KPT-276, KPT-330, and KPT-335, improved cytotoxicity by slowly reversing the covalent bonding [45]. However, this could lead to a decrease in the efficacy of XPO1 inhibitors in clinical applications [46]. Fortunately, the combination of existing chemotherapy drugs and XPO1 inhibitors normally enhanced the efficacy. In CD34-positive myelofibromas, XPO1 inhibitors led to the nuclear accumulation of P53 and enhanced ruxolitinib-mediated cell proliferation, inhibition, and apoptosis [47]. In human non-small cell lung cancer, the combination of selinexor and cisplatin synergistically enhanced the antitumor effects in vitro [39]. Compared with monotherapy, KPT-330 and gemcitabine work synergistically to inhibit pancreatic cancer cells [48]. We observed that KPT-335 monotherapy significantly inhibited the proliferation of neuroblastoma both in vitro and in vivo. In addition, the tumor volume of nude mice was significantly reduced without major organ damage after treatment with KPT335. However, since no clinical study has evaluated the effectiveness of second-generation XPO1 inhibitors in neuroblastoma, we could not speculate on the clinical value of KPT-335 monotherapy in neuroblastoma. We also observed that PI3K agonist 740Y-P partially rescues the in vitro antitumor effects of verdinexor. This means that the PI3K/AKT pathway was involved in the transportation of target proteins by XPO1. Inhibitors of the PI3K/AKT pathway was expected to enhance this effect. Therefore, the combination of verdinexor with PI3K/AKT pathway inhibitors could be promising administration strategy. The efficacy of combining KPT-335 with other chemotherapy drugs as treatment for neuroblastoma should be evaluated in future studies.

## Conclusions

In summary, our study demonstrated that the overexpression of XPO1 was significantly associated with poor clinical characteristics and prognosis in neuroblastoma patients. Inhibition of XPO1 by the specific inhibitor verdinexor suppressed neuroblastoma cell growth and induced cell apoptosis through FOXO1 and RB1 nuclear accumulation by inhibiting the PI3K/AKT pathway. Verdinexor also induced P53 nuclear accumulation and induced G0/G1 phase cell cycle arrest in neuroblastoma cells by activating P53 function. In an in vivo study, verdinexor inhibited tumor growth without obvious major organ damage. Therefore, XPO1 is a promising prognostic indicator for neuroblastoma and represents a novel therapeutic target by the selective inhibitor verdinexor. Further work could focus on the efficacy of a combination of verdinexor with other chemotherapy drugs in neuroblastoma.

## Supplementary Information

**Additional file 1:Figure S1.** The knockdown efficiency of siRNAs. (A) siRNAs reduced XPO1 protein expression. (B) siRNA reduced FOXO1 and RB1 protein expression. (C) siRNA reduced P53 protein expression.

## Data Availability

The datasets supporting the conclusions of this article are available in the Gene Expression Omnibus repository, [GSE85047, https://www.ncbi.nlm.nih.gov/geo/query/acc.cgi?acc=GSE85047; GSE163987, https://www.ncbi.nlm.nih.gov/geo/query/acc.cgi?acc = GSE163987].

## Abbreviations

NRC: Neuroblastoma Research Consortium
XPO1: Exportin-1
CRM1: Chromosomal region maintenance 1
SINE: Selective inhibitors of nuclear transport
TMA: Tissue microarray
CCK-8: Cell Counting Kit-8 reagent
PBS: Phosphate buffer saline
PI: Propidium iodide
TUNEL: TdT-mediated dUTP nick-end labeling
SDS-PAGE: Sodiumdodecyl sulfate–polyacrylamide gel electrophoresis
IF: Immunofluorescence
IHC: Immunohistochemistry
H-score: Histologic scoring system
INSS: International Neuroblastoma Staging System
LMB: Leptomycin B
