# Supplementary figures and images for: XPO1/CRM1 is a promising prognostic indicator for neuroblastoma and represented a therapeutic target by selective inhibitor verdinexor
Source: J Exp Clin Cancer Res. 2021 Aug 12;40:255. doi: 10.1186/s13046-021-02044-z (PMC8359549; doi:10.1186/s13046-021-02044-z)

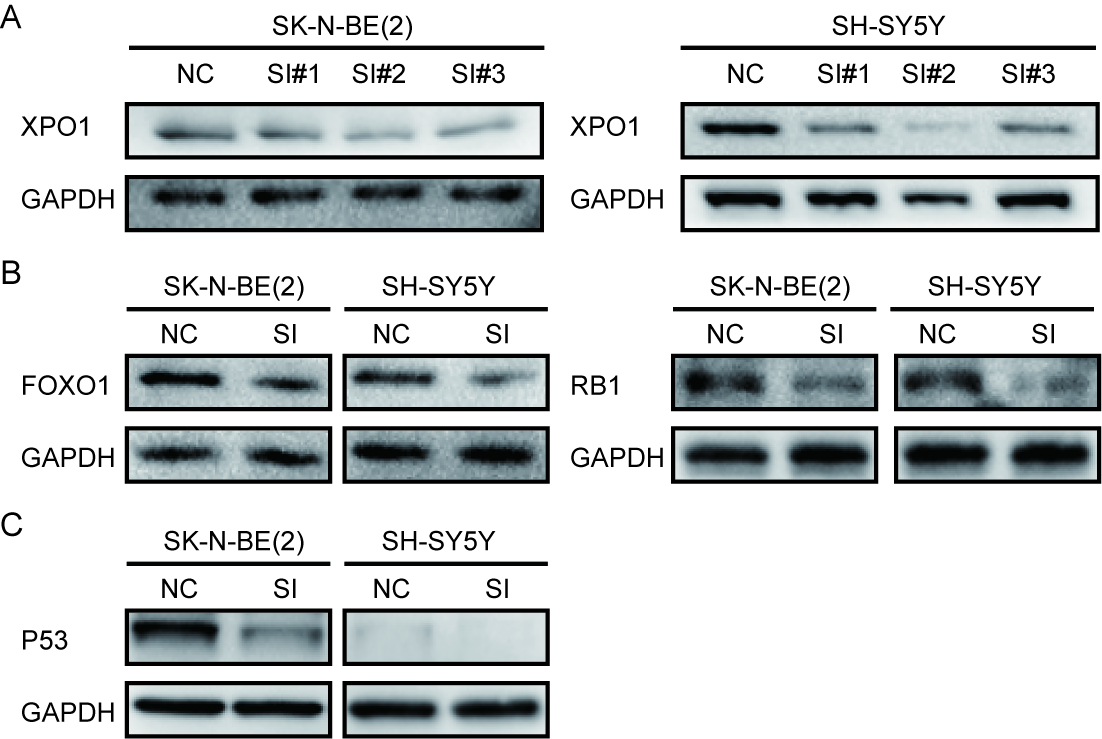

Supplement: Supplementary file 1 — Additional file 1:Figure S1. The knockdown efficiency of siRNAs. (A) siRNAs reduced XPO1 protein expression. (B) siRNA reduced FOXO1 and RB1 protein expression. (C) siRNA reduced P53 protein expression. [file 13046_2021_2044_MOESM1_ESM.tif]
